# Results after Cementless Medial Oxford Unicompartmental Knee Replacement - Incidence of Radiolucent Lines

**DOI:** 10.1371/journal.pone.0170324

**Published:** 2017-01-19

**Authors:** Benjamin Panzram, Ines Bertlich, Tobias Reiner, Tilman Walker, Sébastien Hagmann, Marc-André Weber, Tobias Gotterbarm

**Affiliations:** 1 Clinic of Orthopedic and Trauma Surgery, University of Heidelberg, Heidelberg, Germany; 2 Clinic of Diagnostic and Interventional Radiology, University of Heidelberg, Heidelberg, Germany; Georgia Regents University, UNITED STATES

## Abstract

**Purpose:**

Tibial radiolucent lines (RL) are commonly seen in cemented unicompartmental knee replacement (UKR). In the postoperative course, they can be misinterpreted as signs of loosening, thus leading to unnecessary revision. Since 2004, a cementless OUKR is available. First studies and registry data have shown equally good clinical results of cementless OUKR compared to the cemented version and a significantly reduced incidence of RL in cementless implants.

**Methods:**

This single-centre retrospective cohort study includes the first 30 UKR (27 patients) implanted between 2007 and 2009 with a mean follow-up of 5 years. Clinical outcome was evaluated using the OKS, AKS, range of movement (ROM) and level of pain (VAS). Standard radiologic evaluation was performed at three months, one and five years after surgery. The results five years after implantation were compared to a group of 27 cemented Oxford UKR (OUKR) in a matched-pair-analysis.

**Results:**

Tibial RL were seen in 10 implants three months after operation, which significantly decreased to five after one year and to three after five years (p = 0.02). RL did not have a significant influence on revision (p = 1.0) or clinical outcome after five years. RL were always partial, never progressive and strictly limited to the tibia. There was no significant difference in the incidence of tibial RL five years after implantation between cemented and cementless implants (cemented: 4, cementless: 3, p = 1.0).

**Conclusions:**

After cementless implantation RL were limited to the tibia, partial and never progressive. During short term follow-up the incidence of RL decreased significantly. RL seem to have no influence on clinical outcome and revision.

## Introduction

Cemented medial unicondylar knee replacement is the gold standard for the therapy of anteromedial osteoarthritis of the knee. It has proven excellent long-term survival rates and has many advantages compared to total knee replacement such as smaller incision, minor loss of blood, preservation of bone stock and physiological knee kinematics, shorter hospital stay and rapid recovery.[1–3] The main causes of revision in national joint registries are aseptic loosening of the implant and pain.[4, 5] When pain is associated with physiological radiolucent lines, it might be misinterpreted as a sign of loosening and thereby may lead to unnecessary revisions. [6]

Physiological RL are usually <2 mm thick, have a dense sclerotic margin, are mostly partial and develop during the first year after implantation, without further progress. Physiological RL are considered not to be associated with poor clinical outcome or increased complications.[6, 7] Pathological RL, in contrast, are thicker, do not have a well-defined border, are progressive and associated with loosening of the implant.[7]

A decrease of physiological RL might therefore lower revision rates in cementless OUKR.[8] Cemented OUKR show physiological RL in 40–60% of cases. [6, 9, 10]

Since 2004, a cementless fixation system of the OUKR is available. First studies indicate comparable short- and medium-term clinical outcome and data from the New Zealand Joint Registry indicated a decrease of revision rates of up to 50% in cementless OUKR. [8, 11, 12]

The aim of this study is to assess the incidence and the progression of RL after cementless OUKR and the comparison to a group of cemented OUKR in an independent series.

## Patients and Methods

This single-centre cohort study includes the first 30 cementless OUKR (27 patients), consecutively implanted between 2007 and 2009 in our clinic.

The study was conducted in accordance with the Helsinki Declaration of 1975, as revised in 2013. The institutional review board of the University of Heidelberg approved all procedures of this study (S-546/2013). Written consent was obtained from all patients included in this study.

The cementless implantation was performed by three consultant orthopedic surgeons experienced in the Oxford Phase III unicompartmental knee prosthesis (Biomet UK Ltd, Swindon, United Kingdom)

All patients included in this study suffered from anteromedial osteoarthritis (AMOA). In all Patients surgery was indicated using the Oxford criteria. [13, 14] The final decision about surgery was taken intra-operatively by the surgeon. The surgical technique has been described elsewhere using the Oxford Phase III instrumentation.[15]

### Implant Design and Implantation

Few changes were made by the developing centres to allow cementless implantation: All surfaces in contact with bone except for the vertical wall and the femoral pegs are coated with porous titanium and calcium hydroxylapatite. The cut for the tibial keel is slightly narrower than in the cemented model to ensure a tight press-fit and the femoral component is implanted in a more flexed knee position, so that it extends a further 17° anteriorly. The femoral component has two cylindrical HA-coated pegs to impede rotational stress on the implant.[8, 12]

After surgery, patients were guided towards early weight bearing of the knee with consecutive rehabilitation of three weeks. Clinical and radiological follow-up was performed three months, one- and five years after implantation. Clinical outcome was analyzed using range of motion (ROM), pain scale (VAS), American Knee Society Score (AKS) and Oxford Knee Score (OKS).

Revision for any cause and complications were recorded at each follow-up assessment. Revision was defined as the removal or replacement of any of the components.

**Fig 1:**
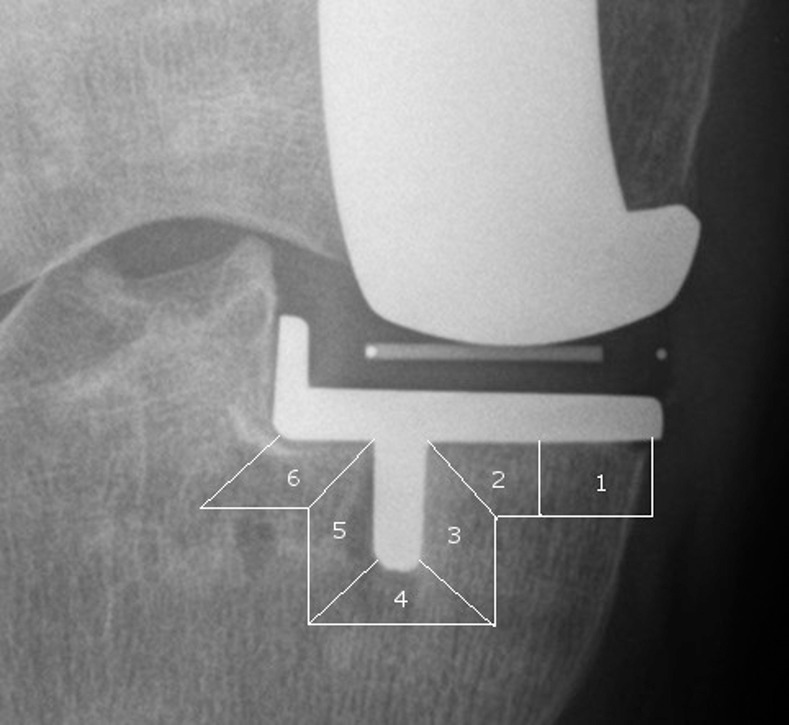
Zones of radiolucent lines on an a.p. radiograph.

**Fig 2:**
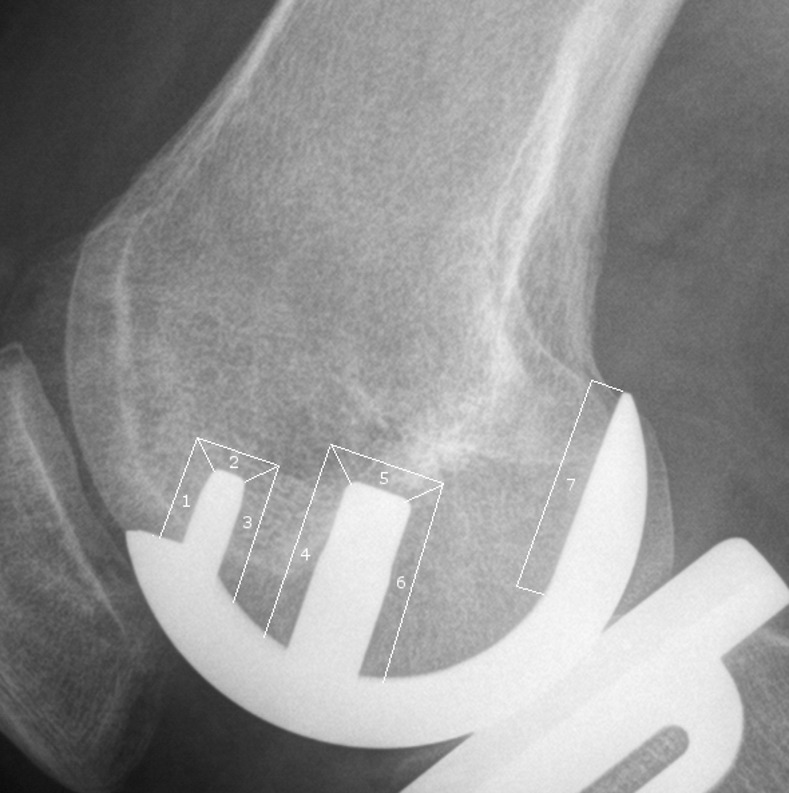
Zones of radiolucent lines on a lateral radiograph.

The radiographs were recorded according to standard protocol as described by Gulati et al.[6] on a digital radiography system consisting of X-ray tube (SRO 33,100), generator (Optimus 50) and digital flat panel detector (“Digital Diagnost”, all Philips Healthcare, Best, The Netherlands) with the patient in a lying position. The x-ray source was aligned to the tibial and femoral components using fluoroscopically guided radiographs. The images obtained in this way were sent to a picture archiving and communications system (PACS) workstation (Centricity^®^ PACS 4.0, GE Healthcare, Barrington, Illinois). Tibial RL were assessed by two independent observers on the AP radiograph, dividing the weight-bearing zones underneath the implant into six zones in cementless and cemented fixation (Fig 1).[6] The vertical wall of the implant is considered non-weight-bearing and therefore is not included in the analysis of RL.[6, 12] Femoral RL were assessed in the lateral radiograph and divided into six zones in case of cementless implantation and four zones in cemented models (Fig 2).[16]

In a matched-pair-analysis, the incidence of radiolucent lines was compared to a group of 27 patients with cemented OUKR that were implanted at the same hospital between 2001 and 2009. Matching criteria were sex, age, BMI and preoperative OKS Score.

### Statistics

Data was described and analyzed using IBM SPSS Statistics, version 21 (SPSS Inc., Somers, NY). To compare repeated measurements on a single sample, Wilcoxon signed-rank test was used. Mann-Whitney U test was chosen to compare differences between two independent groups with an ordinal or continuous dependent variable. Categorical or ordinal variables were counted and tested with Pearson’s Chi^2^ and McNemar test to detect differences. Significance levels were approved at α = 0.05 or smaller.

## Results

The mean follow-up of this study was 60.0 months (range, 47–69; SD, 8.3) and included 30 cementless OUKR from 27 patients (15 male, 12 female). Mean age at surgery was 62.5 years (range, 49–76; SD, 8.3).

No patient died or was lost to follow-up. Three knees were excluded from the study: One knee was excluded because of significant deviation from the surgical technique recommended by the manufacturer. There was one case of periprosthetic tibial plateau fracture within the first month after implantation which was revised to a cemented tibial component and ORIF. The second patient was revised to total knee replacement 26 months after implantation due to progressive OA of the lateral compartment and the patellofemoral joint (PFJ). Additionally, there were two cases with reoperations: One case of mobile-bearing dislocation that occurred 21 months after surgery. The 4 mm bearing was exchanged to a 5 mm one. Another reoperation was performed due to OA of the PFJ. The patient was additionally provided with a patellar-femoro arthroplasty without exchange of the previously implanted components.

The cementless group did not show femoral RL at any time. Whereas one patient in the cemented group developed RL in zone 1 and 3 of the femoral implant. Three months after implantation, 10 knees showed partial RL around the tibial component. All tibial RL in the cementless group were partial and developed within the first three months after surgery. The incidence of RL decreased significantly to five RL at one-year follow-up and three RL at five-year follow-up (p = 0.016, Table 1).

**Table 1:** Radiolucent lines in the cementless group in the course of time.

	*3 months after surgery* ^*a*^	*1 year after surgery*	*5*^*a*^ *years after surgery* ^*a*^
*Case 1*	RL	-	-
*Case 2*	RL	-	-
*Case 3*	RL	RL	RL
*Case 4*	RL	-	-
*Case 5*	RL	-	RL
*Case 6*	RL	RL	RL
*Case 7*	RL	RL	-
*Case 8*	RL	RL	-
*Case 9*	RL	-	-
*Case 10*	RL	RL	-

^a^ decrease of RL from 3 months to 5 years, p = 0.016

**Fig 3:**
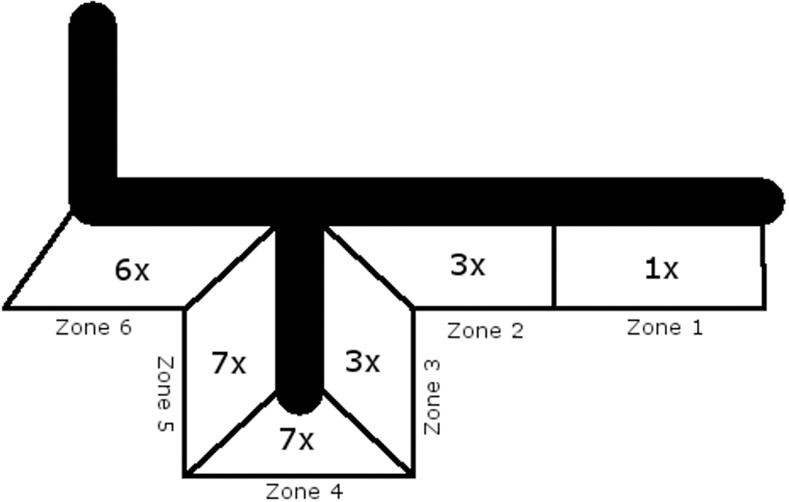
Zones affected by RL 3 months after surgery, cementless.

**Fig 4:**
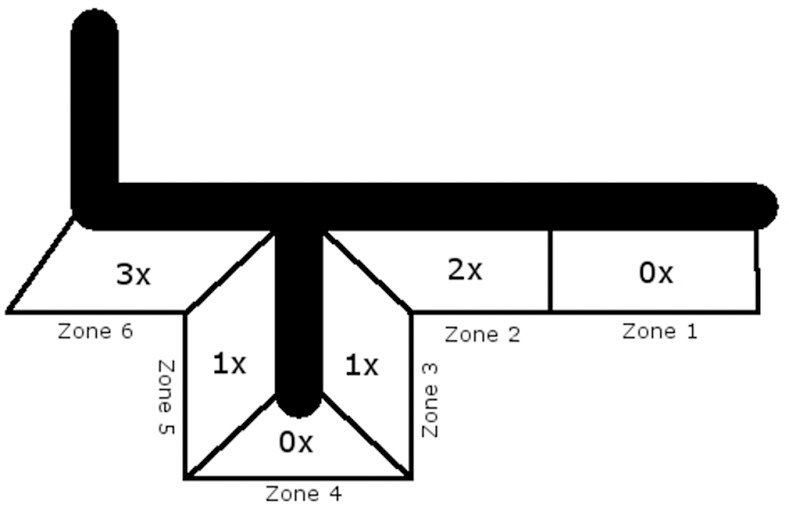
Zones affected by RL 5 years after surgery, cementless.

Three months after implantation, each zone was affected by RL at least once, with the zones four, five and six having the highest incidence of RL (zone four: 7; zone five: 7; zone six: 6), (Fig 3). Five years after implantation, there were RL in zones two, three, five and six (Table 2 and Fig 4). The overall incidence was highest in zone six. In the cemented group, patients with RL tended to be affected in more zones than in the cementless group (2.3 vs. 2.75 zones).

**Table 2:** Tibial RL five years after surgery.

	*Zone 1*	*Zone 2*	*Zone 3*	*Zone 4*	*Zone 5*	*Zone 6*
***Cementless***	**0**	**2**	**1**	**0**	**1**	**3**
*Patient 1*	0	1	0	0	0	1
*Patient 2*	0	0	0	0	1	1
*Patient 3*	0	1	1	0	0	1
***Cemented***	**4**	**3**	**1**	**1**	**1**	**1**
*Patient 1*	1	1	0	0	0	0
*Patient 2*	1	0	0	0	0	0
*Patient 3*	1	1	1	1	1	1
*Patient 4*	1	1	0	0	0	0

There was no significant difference in the clinical outcome regarding ROM, AKS, OKS or pain (VAS) between patients with and without RL 5 years after surgery (Table 3).

**Table 3:** Scores of patients with and without RL in the cementless group five years after implantation.

	*RL (mean)*	*No RL (mean)*	*P-value*
*AKS post-op*	97.0	92.2	0.799
*Δ AKS*	43.0	41.9	0.799
*ROM post-op*	135.0	128.1	0.393
*Δ ROM*	20.0	6.5	0.084
*OKS post-op*	13.0	18.5	0.313
*Δ OKS*	17.7	14.6	0.393
*VAS post-op*	0.7	0.9	1.0
*Δ VAS*	6.0	6.1	0.856

Patients with and without RL did not show significant differences regarding revision or reoperation of their implants (p = 1.0). None of the three implants with RL after five years had to be revised (Table 4). Patient-related factors such as age (p = 0.726), BMI (p = 0.673) or frequency of sports (p = 0.393) did not have any significant influence on the appearance of RL.

**Table 4:** Revision and reoperation in patients with and without RL in the cemented group five years after surgery. Cross tabulation.

	*RL*	*No RL*	*Total*
*Revision or Reoperation*	0	2	2
*No Revision*	3	22	25
***Total***	3	24	27

The matched-pair-analysis was well matched (Table 5). In the cemented group there were 4 tibial RL after five years, of which one was complete, and one partial femoral RL. Overall, there was no significant difference between cemented and cementless implantation concerning the incidence of femoral (p = 1.0) or tibial (p = 1.0) RL five years after implantation. Incidence and classification of RL in both groups are illustrated in Table 6. Between the cemented and the cementless group, no significant disproportion was observed in the incidence of the affected zones.

**Table 5:** Matching-criteria for the cemented and cementless patient group.

	*Cementless*	*Cemented*	*P-value*
*Sex [m/w] (SD)*	15/12	15/12	1.0
*Age [years] (SD)*	62.4 (7.5)	61.4 (7.6)	0.685
*BMI [kg/m^2^] (SD)*	28.0 (3.7)	27.9 (4.5)	0.993
*OKS pre-s*. *[points] (SD)*	32.9 (6.4)	33.1 (8.4)	0.903

**Table 6:** Matched-pair analysis: Radiolucent lines in the cementless vs. cemented group five years after surgery.

	*Tibial RL*				*Femoral RL*			
	*N*^*o*^ *of radiographs*	*N*^*o*^ *of RL*	*Partial*	*Complete*	*N*^*o*^ *of radiographs*	*N*^*o*^ *of RL*	*Partial*	*Complete*
*Cementless*	27	3	3	0	27	0	0	0
*Cemented*	27	4	3	1	27	1	1	0

## Discussion

This single-centre cohort study assessed the incidence of RL and their influence on clinical outcome in the first 27 consecutive patients (15 male, 12 female, 30 knees) that were treated with cementless, medial OUKR in our institution between 2007 and 2009. Mean follow-up time was 60.0 months (47–69; SD 8.3) and mean age at surgery was 62.5 years (range 49–76).

Currently, there is not much data about cementless OUKR apart from the designing centres and joint registries. Recent studies showed a significantly lower incidence of RL in cementless than in cemented UKR. The largest study concerning the cementless Oxford system was published by Liddle et al. with 1.000 implants and showed a RL-rate of 8.9%. Hooper et al. detected only three RL in 196 knees within the first two years after implantation. The prospective randomized trial by Pandit et al. showed only 7% partial tibial RL in the cementless group compared to 75% (43% partial) in the cemented group, of which 32% were complete RL. [8, 12, 16]

In our study we did not find a significant difference concerning the incidence of RL in cementless and cemented fixation (3/27 versus 4/27). Nonetheless, there was one partial femoral RL and one complete tibial RL in the cemented group, while the cementless group only showed partial tibial RL. These patients with RL did neither have any failure nor a poorer clinical outcome. The incidence of RL in the cemented group was much lower than in common literature findings (40–100%).[6, 9, 10] In this context, the role of jet-lavage, which is being used regularly for cemented implantation in our clinic, needs to be taken into account. Clarius et al. showed that the use of jet-lavage in cemented fixation significantly lowers the incidence of RL.[17] This could explain the low rates of RL in our cemented group and might have contributed to the fact that we did not observe a significantly lower rate of RL in the cementless group.

The relatively high rate of RL in 3/27 cementless knees (11%) could be favored by the small number of patients included in this study. Although the designing centres have reported very low rates of RL, our study lies within the average range of RL in cementless implantation, which varies between 1.5% and 17%.[8, 18–20]

The incidence of RL decreased significantly from 10 RL three months after implantation to five RL after one year and three RL five years after surgery (p = 0.002). A reduction of RL within the first year seems to be typical in cementless fixation which can be observed in other studies of cementless OUKR.[8, 20–22]. As explained by Hooper et al. and Pandit et al., early radiolucencies might be ascribed to incompletely impacted implants which settle during weight-bearing. [16, 21] These results, along with the findings by Liddle et al., support our outcome that no new RL were detected later than three months after implantation. As hypothesized in other studies, a follow-up interval of one year seems to be sufficient to evaluate primary fixation of the prosthesis.[12, 23]

In trials analyzing the zones affected by RL, the highest incidences of RL are usually found in zone one and six.[6, 12, 16] In this study, zone six also shows the highest rate of RL (three cases within five years after implantation). Reasons for a possible affection of this zone may be due to different loads, properties of implant-bone-contact or knee kinematics and need to be assessed in further studies.

The presence of RL did not depend on demographic factors such as age, BMI or frequency of sports before or after surgery and did not seem to have a negative impact on the clinical outcome or the implant survival. These findings are in line with other studies assessing the influence of physiological RL on the outcome and survival. [6, 9, 12]

In accordance to other studies, there was no significant difference in the survival rates between cemented and cementless implantation. [8, 12, 24]

The main limitations of this study are the small number of cases and the retrospective single-centre approach. Furthermore, the evaluation of RL was performed by two persons, so that there is still a risk of observer-dependent variability in the results. When evaluating our results, we need to be aware that we investigated the first 30 knees, consecutively operated in our clinic. Thus, selection bias is highly probable. Furthermore, the learning curve of the surgeon needs to be taken into account, especially regarding the periprosthetic tibial plateau fracture. In literature this rare but serious complication reported in cementless fixation has also been described and attributed to mistakes in operative technique.[12, 25]

Regarding the relation between revision and RL, it has to be considered, that two of the three revisions in this patient group were performed before the follow-up radiographs were taken. They were revised to a full or partial cemented prosthesis and thus could not be included in this trial, which might have caused a bias in the relation of revision and RL.

The main strength of this study is the relatively long follow up compared to other literature and that no patient died or was lost during that period of time. This study is among the first published trials for cementless OUKR with a five-year follow-up and describing the prevalence of RL over this time period.

## Conclusion

RL after cementless OUKR implantation demarcate within the first months after operation and significantly regress over time. They are limited to the tibia, partial and do not progress. As important notion, they seem to have no influence on clinical outcome and revision.
